# The combined effect of Parathyroid hormone (1-34) and whole-body Vibration exercise in the treatment of OSteoporosis (PaVOS)- study protocol for a randomized controlled trial

**DOI:** 10.1186/s13063-018-2551-5

**Published:** 2018-03-16

**Authors:** Ditte Beck Jepsen, Jesper Ryg, Niklas Rye Jørgensen, Stinus Hansen, Tahir Masud

**Affiliations:** 1 Department of Geriatric Medicine, Odense University Hospital, Odense, Denmark; 2 Institute of Clinical Research, Faculty of Health Science, University of Southern Denmark, Odense, Denmark; 3 Department of Clinical Biochemistry, Rigshospitalet, Copenhagen, Denmark; 4 OPEN – Odense Patient data Explorative Network, The University of Southern Denmark, Odense, Denmark; 5 Department of Endocrinology, Odense University Hospital, Odense, Denmark; 6 Department of Geriatric Medicine, Nottingham University Hospitals Trust NHS, Nottingham, UK

**Keywords:** Osteoporosis, Whole-body vibration, Parathyroid hormone, Teriparatide, Bone mineral density, Bone quality, RCT

## Abstract

**Background:**

PaVOS is a randomized controlled trial (RCT) which aims to address the use of whole-body vibration exercise (WBV) in combination with parathyroid hormone 1-34 fragment teriparatide (PTH 1-34) treatment in patients with osteoporosis. PTH 1-34 is an effective but expensive anabolic treatment for osteoporosis. WBV has been found to stimulate muscle and bone growth. Animal studies have shown a beneficial effect on bone when combining PTH 1-34 with mechanical loading. A combined treatment with PTH 1-34 and WBV may potentially have beneficial effects on bone and muscles, and reduce fracture risk.

**Methods/design:**

PaVOS is a multicenter, assessor-blinded, superiority, two-armed randomized controlled trial (RCT).

Postmenopausal women (*n* = 40, aged 50 years and older) starting taking PTH 1-34 from outpatient clinics will be randomized and assigned to a PTH 1-34 + WBV-exercise group (intervention group), or a PTH 1-34-alone group (control group).

The intervention group will undergo WBV three sessions a week (12 min each, including 1:1 ratio of exercise: rest, 30 Hz, 1 mm amplitude) for a 12-month intervention period. Both the intervention and the control group will receive PTH 1-34 treatment (20 μg s.c. daily) for 24 months. After 12 months the WBV group will be re-randomized to stop or continue WBV for an additional 12 months.

The primary endpoint, bone mineral density (BMD), will be measured by dual-energy x-ray absorptiometry of the total hip and the lumbar spine.

Secondary endpoints, bone microarchitecture and estimated bone strength, will be assessed using high-resolution peripheral quantitative computed tomography (HR-pQCT) of the radius and tibia. Serum bone turnover markers (carboxy-terminal collagen crosslinks (CTX), amino-terminal propeptide of type-I collagen (P1NP), and sclerostin) and functional biomarkers (Timed Up and Go (TUG), Short Physical Performance Battery (SPPB), grip strength, and leg extension power) will be measured to assess the effect on bone turnover, muscle strength, balance, and functionality. Quality of life (EQ-5D), physical activity (IPAQ) and fear of falling (FES-I) will be assessed by questionnaires. Data on adherence and falls incidence will be collected.

**Discussion:**

The PaVOS study will investigate the effects of WBV in combination with PTH 1-34 on bone parameters in postmenopausal women.

**Trial registration:**

ClinicalTrials.gov, ID: NCT02563353. Registered on 30 September 2015.

**Electronic supplementary material:**

The online version of this article (10.1186/s13063-018-2551-5) contains supplementary material, which is available to authorized users.

## Background

### Introduction

Osteoporosis is defined by the World Health Organization (WHO) as a disease with low bone mass and impaired microarchitecture leading to a high risk of fragility fractures [1].

Fragility fractures of the hip are associated with significant morbidity, institutionalization, and a 1-year mortality of > 20% [2]. Vertebral fractures are also associated with osteoporosis and can lead to significant pain, disability, morbidity, and reduced quality of life [3]. Osteoporotic fractures cause a significant disease burden in developed countries [4] and a recent study of the societal burden imposed by osteoporotic fractures in Denmark showed an estimated yearly cost of €1.563 billion [5].

The parathyroid hormone 1-34 fragment teriparatide (PTH 1-34) is the most commonly used anabolic agent for the treatment of osteoporosis. The Fracture Prevention Trial showed that PTH 1-34 significantly reduced the incidences of vertebral and non-vertebral fractures [6]. However, the relatively high cost of PTH 1-34 (€7103 per year) restricts its use in osteoporotic patients to those with the highest fracture risk or, those who had inadequate response to, or cannot tolerate more commonly used anti-osteoporotic agents, such as bisphosphonates [7]. Thus, any intervention that can boost the efficacy of PTH 1-34 could make it more cost-effective. Furthermore, as the length of PTH 1-34 treatment is limited (licensed for up to 18–24 months only), and maximizing its response by such augmentation would be desirable, in terms of reducing fracture risk in individual patients and by potential cost-saving implications.

Mechanical loading is also known to increase bone formation and load-bearing exercise is an important component in maintaining and improving bone health. Studies have shown that load-bearing exercise can increase bone mineral density (BMD) [8].

A synergistic or additive effect of combined PTH 1-34 treatment and mechanical loading has been reported [9, 10]. Not all older people, however, can undertake high-intensity or weight-bearing exercise. A proposed alternative is whole-body vibration (WBV) therapy which, like weight-bearing exercise, stimulates muscles and bones. In some studies, WBV increases the anabolic (bone-building) effects in bone tissue, as well as increasing BMD [11, 12]. One hypothesis suggests that the effects of vibration directly activate mechanosensors in bone cells [13]. Like weight-bearing exercise [14], WBV may thus improve muscle strength and power by increasing neuromuscular activation [15]. In healthy volunteers, the effects of vibration therapy on muscle strength and performance were similar to those of short-term resistance exercise [14].

WBV therapy may also improve blood circulation in muscle and bone and increase the supply of nutrients needed to build bones [16]. Platforms for WBV are now commercially available and are used in gyms, as an alternative form of exercise. Animal studies with the combination of PTH analogs and WBV are few and with diverse results. One study with low-magnitude vibration and PTH 1-34 in mice showed no synergy in increasing bone mineral content after 8 weeks of treatment [17]. A recently published study showed that the combination of noise-like WBV and PTH treatment of fracture healing produced an additive effect in increasing bone formation and enhancing the mechanical function of the bone [18]. Human studies have shown that vibration can be anabolic to bone [12, 19, 20], and a study performed by one of our group (TM) has shown that WBV is well tolerated even by frail, older individuals and increases muscle strength and bone-formation markers [19]. Adding mechanical loading in the form of WBV to the treatment with an anabolic agent may improve treatment outcomes further increasing BMD and reducing the risk of fracture.

#### Study objectives

To determine if WBV in addition to standard PTH 1-34 treatment has a greater effect in osteoporotic patients compared to standard PTH 1-34 treatment alone on:

- BMD
- Bone microarchitecture
- Markers of bone formation and resorption
- Muscle function and balance
- To assess the safety and adherence to WBV in osteoporotic patients

## Methods/design

### General design

**Fig. 1:**
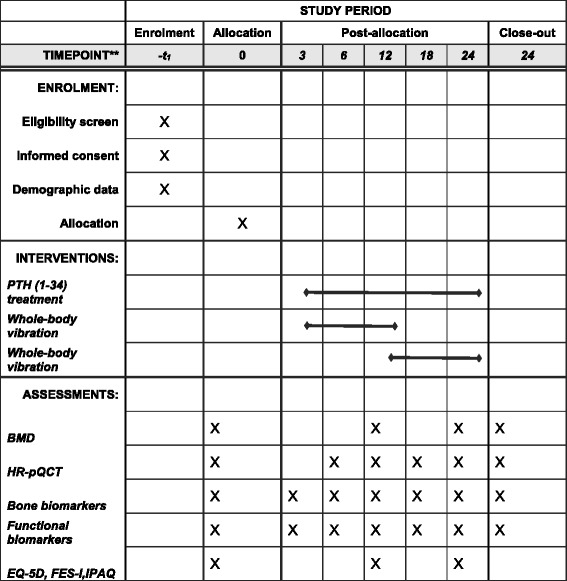
The Standard Protocol Items: Recommendations for Interventional Trials (SPIRIT) Figure with the schedule of enrollment, interventions, and assessments. *BMD* bone mineral density; *Bone turnover biomarkers* procollagen type-1 amino-terminal propeptide, carboxy-terminal type-1 collagen crosslinks, sclerostin; *HR-pQCT* high-resolution peripheral quantitative computed tomography; *FES-I* Falls Efficacy Scale International; *Functional biomarkers*: Short Physical Performance Battery, Timed Up and Go, grip strength, leg extension power; *IPAQ* International Physical Activity Questionnaire.

This will be a multicenter, assessor-blinded, superiority, two-armed RCT in osteoporotic patients starting standard PTH 1-34 treatment. In Denmark, PTH 1-34 is used for the treatment of severe osteoporosis, and the cost is reimbursed if patients have a lumbar spine or total hip T-score ≤ − 3 combined with at least one clinical vertebral fracture (compression ≥ 25%) within the past 3 years or at least two vertebral fractures (compression ≥ 25%) independently of BMD. Participants will be randomized to PTH 1-34 treatment alone or to combined PTH 1-34 treatment and WBV (see Fig. 1). The study will be reported according to the Consolidated Standards of Reporting Trials (CONSORT) extension to non-pharmaceutical interventions and the Standard Protocol Items: Recommendations for Interventional Trials (SPIRIT) 2013 Checklist for RCTs (Additional file 1).

### Participants

Postmenopausal women over the age of 50 years attending either of the following outpatient clinics will be recruited: the Osteoporosis Clinics at Odense University Hospital (Svendborg or Odense), the Department of Geriatrics at Odense University Hospital, the Department of Endocrinology at Hospital of Southwest Denmark, the Department of Endocrinology at Hospital Lillebaelt or the Department of Endocrinology and Internal Medicine, Aarhus University Hospital.

#### Inclusion criteria

- Postmenopausal women aged ≥ 50 years starting PTH 1-34 treatment for osteoporosis

#### Exclusion criteria

- Currently taking oral glucocorticoids
- Unable to give informed consent
- Unable to stand for 1 min at a time on the vibration platform
- Contraindications to WBV (e.g., joint prosthesis, pacemakers)

### Intervention

After instructions by nurses in the treating clinics all participants will self-administer subcutaneously (s.c) PTH 1-34 (20 μg s.c. daily) during the study. The participants follow each clinic’s control program including measurements of serum calcium and creatinine throughout the study. Medical records will be used to check the adherence to the PTH 1-34 treatment.

Half of the participants will be randomized to an intervention group, receiving WBW as add-on treatment. WBV will be undertaken using power plate My5 (Power plate®, UK). The power plate machine oscillates in all three planes, with a frequency of 30 Hz and amplitude of 1 mm (low displacement) and peak acceleration of 35.53 ms^-2^ rms (3.6 *g*).

The WBV intervention will be performed three times a week according to a training protocol (Table 1). The vibration platforms will be delivered and installed in the participant’s own home and instructions for use and the training program will be given by one of the investigators (DJ).

**Table 1:** Training protocol

Week	First training day	Second training day	Third training day
1	30 s × 2	30 s × 3	30 s × 4
2	1 min × 1	1 min ×2	1 min × 3
3–5	1 min × 4	1 min × 4	1 min × 4
6–7	1 min × 5	1 min × 5	1 min × 5
8 to the end of study	1 min × 6	1 min × 6	1 min × 6

*Min* minutes, *s* seconds

The WBV intervention will be conducted with the knees slightly bent (at approximately 20°) to prevent vibrations to cause side effect as dizziness from the vibration transmitted to the head. The training period will be followed by a resting period in the ratio 1:1 and the training days will be conducted with a resting day in between.

### Randomization

**Fig. 2:**
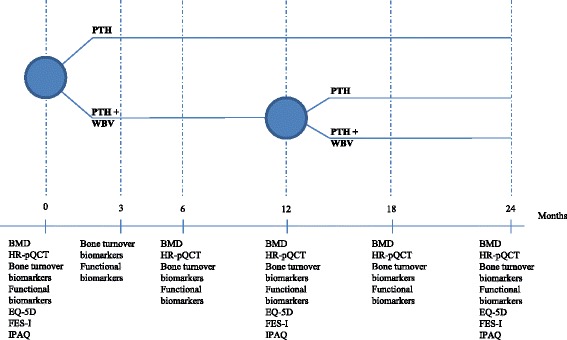
Timeline for participants. Figure 2 shows the timeline for the participants in the study. The *x*-axis is the time during the study in months. The participants will be randomized after baseline data is collected to an intervention group receiving whole-body vibration (WBV) in addition to PTH 1-34 compared to the control group receiving PTH 1-34 alone. After 12 months the intervention group will be re-randomized. *BMD* bone mineral density; *HR-pQCT* high-resolution peripheral quantitative computed tomography; *Bone biomarkers* procollagen type-1 amino-terminal propeptide (P1NP), carboxy-terminal type-1 collagen crosslinks (CTX), and sclerostin; *Physical markers*: Timed Up and Go (TUG), Short Physical Performance Battery (SPPB), grip strength and leg extensor power; *DXA* dual-energy x-ray absorptiometry; *IPAQ* The International Physical Activity Questionnaire; *FES-I* Falls Efficacy Scale International

After informed consent and collection of baseline data, participants will be randomized into two groups: PTH 1-34 treatment alone or PTH 1-34 treatment + WBV (Fig. 2). The randomization is a web-based, computer-generated block randomization with no stratification. Block size is created by a data manager and the size is unknown to the investigators until the end of the study. Allocation will be concealed to the participants and the investigators until after collection of baseline data.

After 12 months the combined PTH 1-34 + WBV group will be re-randomized to continuation of combined PTH 1-34 + WBV for another 12 months, or to PTH 1-34 alone (i.e., stopping WBV) for another 12 months. This will allow us to determine if any positive effects of WBV wear off after it has been stopped or if any gains persist.

### Endpoints

#### Primary endpoints

The primary endpoint is the percent change in areal BMD of total hip and lumbar spine as measured by dual-energy x-ray absorptiometry (DXA) (Hologic Discovery, Waltham, MA, USA) from baseline until 12 months (Table 2).

**Table 2:** Primary and secondary endpoints

	Measurement	Site	Time (months)
Primary endpoints			
Δ Bone mineral density	DXA	Total hip Spinal region	12
Secondary endpoints			
Δ Bone mineral density	DXA	Total hip Spinal region	6, 18, and 24
Δ Bone microarchitecture	HR-pQCT	Distal radius Distal tibia	6, 12, 18, and 24
Δ Bone biomarkers	P1NP CTX, sclerostin	Serum	3, 6, 12, 18, and 24
Δ Physical biomarkers	TUG SPPB		3, 6, 12, 18, and 24
	Dynamometer	Hand grip	
	Nottingham power rig	Leg extensor	
Δ Quality of life	EQ-5D		12 and 24
Δ Fear of falling	FES-I		12 and 24
Physical activity	IPAQ		0, 12, and 24
Adherence to WBV	Training logbook		Continuously
Falls	Falls calendar		Continuously

*Δ* change from baseline; Time is measured in months; *DXA* dual-energy x-ray absorptiometry; *HR-pQCT* high-resolution peripheral quantitative computed tomography; *P1NP* procollagen type-1 amino-terminal propeptide; *CTX* carboxy-terminal type-1 collagen crosslinks; *TUG* Timed Up and Go; *SPPB* Short Physical Performance Battery; *FES-I* Falls Efficacy Scale International; *IPAQ* The International Physical Activity Questionnaire; *EQ-5D* EuroQoL 5-dimension, 5-level questionnaire, *WBV* whole-body vibration

BMD is chosen as an endpoint since it is an important determinant of fracture risk and is used to diagnose and assess response to treatment in osteoporotic patients [1, 21].

#### Secondary endpoints

##### Bone mass

Change in areal BMD of total hip and lumbar spine is measured by DXA from baseline until 6, 18, and 24 months (Table 2).

##### Bone microarchitecture

Images from high-resolution peripheral quantitative computed tomography (HR-pQCT) (Xtreme CT, Scanco Medical, AG, Brüttisellen, Switzerland), of the non-dominant distal radius and distal tibia (the opposite limb in the presence of a previous fracture) will be obtained to measure bone geometry, cortical morphology, trabecular morphology, and overall biomechanical competence. In in-vitro studies estimated bone strength of the radius using finite element analysis [22, 23] which has been shown to be more closely correlated to observed radius bone strength than areal BMD by DXA [22]. PTH 1-34 treatment has been shown to be associated with an increase in vertebral estimated bone strength [24] and preservation of the estimated bone strength of the radius and hip [25, 26]. The images are obtained at baseline, 6, 12, 18, and 24 months (Table 2). The reported outcomes will be total BMD, cortical thickness, cortical porosity, bone volume per trabecular volume (BV/TV), trabecular number, trabecular thickness, and finite element failure load. The scanning protocol and the image acquisition have previously been described in detail [22, 23].

##### Serum bone turnover markers

Markers of bone formation (procollagen type-1 amino-terminal propeptide (P1NP)) and bone resorption (carboxy-terminal type-1 collagen crosslinks (CTX-1) will be measured by the method of chemiluminescence (iSYS, Immunodiagnostic Systems Ltd., Boldon, England). Sclerostin is analyzed using TECOmedical Human Sclerostin HS ELISA (TECOmedical group, Sissach, Switzerland). Bone turnover is affected by treatment with PTH 1-34, and studies have shown that a rise in the marker of bone formation, P1NP, during PTH 1-34 treatment is a predictor of the increase in BMD [27]. The assessment of bone biomarkers will allow exploration of mechanisms of action of WBV in augmenting PTH 1-34 treatment on bone turnover. The blood samples are taken at baseline, 3, 6, 12, 18, and 24 months (Table 2) after an overnight fast and stored at − 70 °C until analyzed in a central laboratory.

##### Functional biomarkers

Functional biomarkers including measurements of muscle strength, function and balance is measured by: (1) Timed Up and Go (TUG), (2) Short Physical Performance Battery (SPPB), (3) leg extensor power (Nottingham power rig), and (4) grip strength (Smedley dynamometer, TTM, Tokyo, Japan).

Fragility fractures are most often associated with falls [21, 28]. The measurement of lower extremity function and mobility will indicate if WBV has the potential to reduce the risk of falls in osteoporotic patients, which is another important determinant of the risk of fracture, independent on the effects on bone [21]. The TUG is the measurement of functional mobility and is a simple and reliable clinical test used to screen for fall risk in community-dwelling older persons [29]. Lower-extremity function and balance can also be assessed by the SPPB which is a validated test in older adults [30].

Leg extension power is assessed using a leg extensor power rig, (Medical Engineering Unit, School of Biomedical Sciences, University of Nottingham Medical School, Nottingham, UK) reported to have good reliability with no significant differences found between two tests performed 1 week apart (*r* = 0.97; coefficient of variation = 9%) [31]. At each visit the same leg is tested with a series of five measurements from the same starting position. The leg is selected randomly at the first visit. The SPPB and the TUG are described in detail by others [30, 32]. Reduced grip strength is a predictor of fragility fractures independent of BMD [33]. A reliable and valid method is by using a Smedley dynamometer (TTM, Tokyo, Japan). The measurements are done twice with each hand using a protocol described by others [34].

The functional tests will be performed by technicians, who all have been trained by a physiotherapist and all tests are conducted by standardized protocols. The TUG, SPPB, grip strength and leg extension power tests will be performed at baseline, 3, 6, 12, 18, and 24 months (Table 2).

Full-body DXA (Hologic Discovery, Waltham, MA, USA) is performed at baseline, 12, and 24 months to assess muscle mass (Fig. 2).

Sarcopenia is a condition with low skeletal muscle mass leading to decreased muscle strength, and impaired physical function, associated with an increased risk of falls [35]. Sarcopenia is proposed to be assessed by a variety of measurements of muscle mass and function including but not limited to full-body DXA, SPPB, grip strength, and gait speed [36, 37].

##### Questionnaires

The participants’ physical activity will be measured by a validated questionnaire, the International Physical Activity Questionnaire (IPAQ). The participants’ multiples of the resting metabolic rate will be generated (MET-min/week) [38].

Vertebral fractures have been shown to decrease quality of life [3] and the EuroQol 5-dimension, 5-level questionnaire (EQ-5D) is a well-validated measure of quality of life [39, 40].

The patients’ own fear of falling is associated with the risk of falls [41] and the the Falls Efficacy Scale International (FES-I) questionnaire, which is a reliable and validated questionnaire to measure the fear of falling in an older population [42].

The questionnaires IPAQ, EQ-5D, and FES-I will be distributed in paper versions to the participants at baseline, 12, and 24 months.

##### Adherence

Adherence to WBV is ascertained using self-reported adherence in a log book. Dropouts and the reasons for this will be noted. The adherence to the PTH 1-34 treatment is ascertained by asking the patients and by using the prescription database.

##### Side effects

Information on symptoms of dizziness, pain, and falls will be collected. The participants will be handed a calendar to note fall events, defining a fall as “an unexpected event in which the participants come to rest on the ground, floor, or lower level” [43]. Pain will be assessed by the Numeric Rank Score (0 being no pain, and 10 being unbearable pain) and the question about how many days they have felt dizzy during the last week, and falls will be assessed by monthly telephone calls during the first year and then every 3 months for the rest of the trial.

Demographic data, including data on risks factors associated with osteoporosis and fragility factures, will be collected at baseline and thus before randomization.

These factors include Body Mass Index, current and previous height, age, comorbidity, fall history, previous fractures, vitamin D and calcium intake, current, and previous history of medication.

### Statistical analysis

#### Sample size determination

The inclusion of 32 participants (16 in each group) would give the study 80% power to detect a clinically significant additional increase of 22% in BMD with WBV (assuming a 9% increase of BMD in the PTH 1-34-alone group and 11% increase in the combined PTH 1-34 + WBV group, and assuming a standard deviation (SD) of the BMD increase of 2% [12]. Inclusion of 40 participants (20 in each group) will allow for a 20% dropout rate.

#### Statistical methods and data managing

The intervention arm (PTH + WBV) is compared against the control arm for all primary analyses. We will use analysis of covariance (ANCOVA) and “percentage change” for primary outcome adjusting for baseline measurements. For secondary outcomes we will use a chi-squared test for binary outcomes, analysis of variance (ANOVA) and the *T* test for continuous outcomes, and for non-normally distributed continuous outcomes we will use log transformation or Wilcoxon’s match-pairs signed-rank test and the Kruskal-Wallis test.

All *p* values will be reported to four decimal places with *p* values < 0.001 reported as *p* < 0.001. STATA version 14 will be used to conduct analyses. For all tests, we will use two-sided *p* values with an alpha ≤ 0.05 level of significance.

The analyses will be conducted with the intention-to-treat method, and in order to investigate the effect of adherence and withdrawal a per-protocol analysis will be conducted. Missing data will be handled using multiple imputation. Study data is collected and managed using REDCap (Version 6.5.10 – © 2015 Vanderbilt University) electronic data capture tools hosted at the University of Southern Denmark [44]. Sequential study numbers will be allocated to study patients and entered into the recruitment log at the secure web database REDCap.

## Discussion

This is a RCT in osteoporotic patients randomized to PTH 1-34 treatment alone or to combined PTH 1-34 treatment and WBV. The primary and secondary endpoints in the study are chosen because previous studies have shown such markers of bone mass, microarchitecture, and turnover, as well as indices of physical performance, to be associated with osteoporosis or fragility fractures.

Earlier studies have reported WBV as a feasible intervention [12] and in older patients the adherence is reported to be good [19].

The intervention is designed to be of long duration (12–24 months) and the setting at the participants’ own home makes the training easy and highly accessible.

The study has limitations including the non-blinded design. It is our hypothesis that the primary endpoint, the BMD as well as a number of secondary endpoints (bone microarchitecture and bone turnover markers) are not at risk of bias by the open-label design. The functional biomarkers on the other hand could be affected by the non-blinded design but the study personnel performing the tests are blinded to the allocation and the baseline data is collected before randomization.

Measurements of quality of life, physical activity, and the FES-I questionnaire are patient-collected data, resulting in the possibility of bias in the reporting.

To limit the possibility of bias by attention, the participants will receive close to an equal amount of contacts by the investigators because of the “at home training design.”.

The study is partially blinded and is protected against selection bias by central randomization after baseline measurements, randomization via web with no stratification, and by allocation concealment.

It is our hypothesis that the DXA, HR-pQCT, and bone marker measurements are not at risk of performance bias. The physical biomarkers may be prone to performance bias, but the test personnel will be blinded to avoid evaluation bias.

To avoid attrition in both arms and ensure adherence, the participants are contacted by telephone each month during the first year and every third month during the second year. The participants receive a direct telephone number and mailing address to the primary investigator and are encouraged to make contact in case of any questions or events.

Every patient is motivated to attend follow-up regardless of randomization or adherence. The participants do not receive payment but are reimbursed for transport expenses.

The adherence to the training protocol is patient reported data and is in risk of reportingbias. A previous study nevertheless found high correlation with the usage of a training logbook to electronic monitoring in the WBV intervention in older adults (overall intraclass correlation coefficient = 0.96), showing a small risk of bias in the collected data on adherence [45].

A combined treatment of WBV and PTH 1-34 might have synergistic or additive beneficial effects on bone strength, thereby reducing fracture risk and making the treatment more cost-effective. A beneficial effect of WBV on muscles, and subsequently fall risk, may lower the fracture risk even further, resulting in comprehensive fracture prevention.

## Trial status

Recruitment of participants started in November 2015 and will be completed in September 2017. This study has been registered at ClinicalTrials.gov (September, 2015: NCT02563353).

## Additional file

Additional file 1: SPIRIT 2013 Checklist: recommended items to address in a clinical trial protocol and related documents*. (DOC 124 kb)

## Abbreviations

BMD: Bone mineral density
CTX: Carboxy-terminal type-1 collagen crosslinks
DXA: Dual-energy x-ray absorptiometry
FES-I: Falls Efficacy Scale International
HR-pQCT: High-resolution peripheral quantitative computed tomography
IPAQ: International Physical Activity Questionnaire
P1NP: Procollagen type-1 amino-terminal propeptide
PTH 1-34: Teriparatide
RCT: Randomized controlled trial
SPPB: Short Physical Performance Battery
TUG: Timed Up and Go
WBV: Whole-body vibration
